# Draft Genome Sequences of Enterococcus faecalis Strains Isolated from Healthy Japanese Individuals

**DOI:** 10.1128/MRA.00832-19

**Published:** 2019-10-03

**Authors:** Kazuki Tanaka, Tsubasa Watabe, Kumiko Kato, Tomoya Tsukimi, Mitsuhiko P. Sato, Toshitaka Odamaki, Masaru Tomita, Shinji Fukuda

**Affiliations:** a Institute for Advanced Biosciences, Keio University, Tsuruoka, Yamagata, Japan; b Systems Biology Program, Graduate School of Media and Governance, Keio University, Fujisawa, Kanagawa, Japan; c Intestinal Microbiota Project, Kanagawa Institute of Industrial Science and Technology, Kawasaki, Kanagawa, Japan; d Next Generation Science Institute, Morinaga Milk Industry Co., Ltd., Zama, Kanagawa, Japan; e Department of Bacteriology, Faculty of Medical Sciences, Kyushu University, Higashi, Fukuoka, Japan; f Transborder Medical Research Center, University of Tsukuba, Tsukuba, Ibaraki, Japan; g PRESTO, Japan Science and Technology Agency, Saitama, Japan; University of Maryland School of Medicine

## Abstract

Enterococcus faecalis is a common commensal of the intestines of humans and other mammals but is also a frequent cause of serious ailments. Here, we report 14 draft genome sequences of strains of Enterococcus faecalis, a normal inhabitant and Gram-positive bacterium that was isolated from 7 healthy Japanese volunteers.

## ANNOUNCEMENT

Enterococcus faecalis is a common commensal of the intestines of humans and other mammals. There has been an increasing number of multidrug-resistant strains and nosocomial infections of the bacterium due to the frequent use of antibiotics (1), which drives the need to understand the bacterial infection mechanism of the species.

E. faecalis was isolated from 7 healthy Japanese volunteers on a transoligosaccharide (TOS)-propionate agar medium plate (Yakult Pharmaceutical Industry) and anaerobically cultured for 48 h at 37°C. The isolated colonies from each subject were further incubated with de Man, Rogosa, and Sharpe (MRS) liquid medium (Wako) and cultured anaerobically for 24 h at 37°C. Genomic DNA was isolated using DNeasy blood and tissue kits (Qiagen). Sequencing libraries were prepared using the NEBNext Ultra II FS DNA library prep (New England BioLabs) and sequenced with the HiSeq 2500 platform (Illumina) using 150-bp paired-end read technology. The reads were filtered and trimmed using Platanus_trim v1.0.7 (http://platanus.bio.titech.ac.jp/pltanus_trim), and 940,782 to 1,393,444 reads per sample remained. Trimmed reads were assembled with Platanus v1.2.4 (2), with default parameters, and contigs equal to or shorter than 300 bp were discarded with an in-house script (https://github.com/MitsuhikoP/cut_short_fasta). Genome completeness was analyzed using BUSCO v1 (3) on the gVolante Web server (4), and gene annotation was analyzed using DFAST (2), as previously described (5). Identification of E. faecalis was performed with BLAST+ v2.4.0 (6, 7) and pyani v0.2.7 (https://github.com/widdowquinn/pyani). The draft genome sequences of E. faecalis have 2,794,898 to 2,930,781 bp in a total of 13 to 33 contigs, with an *N*_50_ value of 218,789 to 563,424 bp and GC content of approximately 37.5%. The genomes were predicted to contain 2,627 to 2,793 putative coding sequences.

For comparative analysis, GenBank files of 25 E. faecalis strains were downloaded from the NCBI FTP site on 25 April 2019. Nucleotide sequence alignments for core genes were produced using Roary v3.12.0 (8), with a minimum blastp percentage identity of 95, and MAFFT v7.407 (9). A phylogenetic tree was constructed using FastTree v2.1.3 (10) with the general time-reversible (GTR) plus category (CAT) model. The phylogenetic tree was rooted using the phangorn package v2.4.0 and drawn using the ape package v5.3 in R v3.3.3.

**FIG 1:**
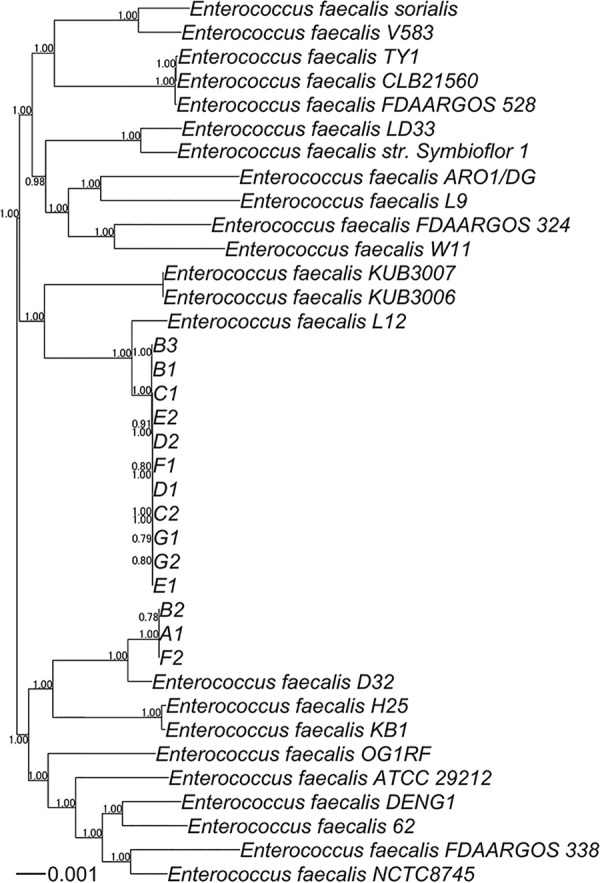
Midpoint rooting phylogenetic tree obtained from a concatenated nucleotide sequence alignment of the 1,849 core genes of the 14 Enterococcus faecalis strains isolated from the 7 volunteers and reference genomes of 25 E. faecalis strains.

The core-genome phylogeny indicated that the clade consisting of the strains isolated from the Japanese volunteers was divided into a clade close to the L12 strain and a clade close to the D32 strain (Fig. 1), which are pig-derived bacteria (GenBank accession numbers CP018102 and CP003726, respectively). Therefore, these strains could be colonized both in humans and in livestock. Furthermore, since a large number of phylogenetically similar strains were also detected from multiple subjects, the strains may be transmitted between humans frequently.

This study was approved by the ethics committee of Keio University Shonan Fujisawa Campus and the Kenshokai under approval numbers 195 and 20170915-4, respectively. All subjects were informed of the purpose of this study, and written consent was obtained from all subjects.

### Data availability.

The GenBank and DDBJ Sequence Read Archive (DRA) accession numbers are listed in Table 1.

**TABLE 1:** Genomic features of strains used in this study

Sample^*a*^	GenBank accession no.	DRA accession no.	GC content (%)^*b*^	No. of contigs	Total contig size (bp)	Largest contig size (bp)	*N*_50_ (bp)	No. of CDSs^*c*^
A1	BJTH00000000	DRR179066	37.5	32	2,928,338	659,735	218,789	2,793
B1	BJTI00000000	DRR179067	37.3	31	2,850,529	890,826	301,644	2,688
B2	BJTJ00000000	DRR179068	37.5	32	2,930,781	659,737	249,285	2,791
B3	BJTK00000000	DRR179069	37.4	29	2,794,898	835,194	318,734	2,627
C1	BJTL00000000	DRR179070	37.5	29	2,871,953	889,827	261,062	2,714
C2	BJTM00000000	DRR179071	37.5	13	2,873,694	1,435,291	563,268	2,271
D1	BJTN00000000	DRR179072	37.5	22	2,872,519	881,255	514,512	2,717
D2	BJTO00000000	DRR179073	37.5	17	2,874,611	889,949	480,305	2,716
E1	BJTP00000000	DRR179074	37.5	18	2,873,047	881,086	563,424	2,716
E2	BJTQ00000000	DRR179075	37.5	19	2,872,883	881,086	562,779	2,713
F1	BJTR00000000	DRR179076	37.5	27	2,873,489	881,203	361,800	2,713
F2	BJTS00000000	DRR179077	37.5	33	2,928,825	659,737	249,285	2,790
G1	BJTT00000000	DRR179078	37.5	25	2,873,406	733,617	424,659	2,712
G2	BJTU00000000	DRR179079	37.5	27	2,874,408	881,085	480,088	2,716

a The sample name represents the subject and the colony number.

b GC content (%) is the relative frequency (percentage) of guanine and cytosine (G+C)/(A+T + G+C).

c CDSs, coding sequences.

## References

[1] Nowakiewicz A, Ziółkowska G, Zięba P, Gnat S, Trościańczyk A, Adaszek Ł 2017 Characterization of multidrug resistant E. faecalis strains from pigs of local origin by ADSRRS-fingerprinting and MALDI-TOF MS; evaluation of the compatibility of methods employed for multidrug resistance analysis. PLoS One 12:e0171160. doi:10.1371/journal.pone.0171160. 28135327 PMC5279778

[2] Kajitani R, Toshimoto K, Noguchi H, Toyoda A, Ogura Y, Okuno M, Yabana M, Harada M, Nagayasu E, Maruyama H, Kohara Y, Fujiyama A, Hayashi T, Itoh T 2014 Efficient de novo assembly of highly heterozygous genomes from whole-genome shotgun short reads. Genome Res 24:1384–1395. doi:10.1101/gr.170720.113. 24755901 PMC4120091

[3] Simão FA, Waterhouse RM, Ioannidis P, Kriventseva EV, Zdobnov EM 2015 BUSCO: assessing genome assembly and annotation completeness with single-copy orthologs. Bioinformatics 31:3210–3212. doi:10.1093/bioinformatics/btv351. 26059717

[4] Nishimura O, Hara Y, Kuraku S 2017 gVolante for standardizing completeness assessment of genome and transcriptome assemblies. Bioinformatics 33:3635–3637. doi:10.1093/bioinformatics/btx445. 29036533 PMC5870689

[5] Evans-Yamamoto D, Takeuchi N, Masuda T, Murai Y, Onuma Y, Mori H, Masuyama N, Ishiguro S, Yachie N, Arakawa K 2019 Complete genome sequence of *Psychrobacter* sp. strain KH172YL61, isolated from deep-sea sediments in the Nankai Trough. Microbiol Resour Announc 8:e00326-19. doi:10.1128/MRA.00326-19. 31000557 PMC6473151

[6] Altschul SF, Gish W, Miller W, Myers EW, Lipman DJ 1990 Basic local alignment search tool. J Mol Biol 215:403–410. doi:10.1016/S0022-2836(05)80360-2. 2231712

[7] Camacho C, Coulouris G, Avagyan V, Ma N, Papadopoulos J, Bealer K, Madden TL 2009 BLAST+: architecture and applications. BMC Bioinformatics 10:421. doi:10.1186/1471-2105-10-421. 20003500 PMC2803857

[8] Page AJ, Cummins CA, Hunt M, Wong VK, Reuter S, Hunt M, Holden MTG, Fookes M, Falush D, Keane JA, Parkhill J 2015 Roary: rapid large-scale prokaryote pan genome analysis. Bioinformatics 31:3691–3693. doi:10.1093/bioinformatics/btv421. 26198102 PMC4817141

[9] Nakamura T, Yamada KD, Tomii K, Katoh K 2018 Parallelization of MAFFT for large-scale multiple sequence alignments. Bioinformatics 34:2490–2492. doi:10.1093/bioinformatics/bty121. 29506019 PMC6041967

[10] Price MN, Dehal PS, Arkin AP 2010 FastTree 2—approximately maximum-likelihood trees for large alignments. PLoS One 5:e9490. doi:10.1371/journal.pone.0009490. 20224823 PMC2835736

